# Can we trust the guidelines? Comparison between the data presented and the recommendations of the International Antiviral Society-USA Panel

**DOI:** 10.7448/IAS.17.4.19766

**Published:** 2014-11-02

**Authors:** Rein Jan Piso

**Affiliations:** Medizinische Klinik, Kantonsspital Olten, Olten, Switzerland

## Abstract

**Introduction:**

Clinicians often do not have the time and possibilities to read all scientific evidence necessary to maintain high quality patient care. They rely on guidelines made by experts to help them in their daily work.

**Methods:**

We compared the 2012 recommendations and the arguments of the IAS-USA Panel with the data referenced and presented with the original data of the studies. Special topic was the timing of antiretroviral therapy. Only studies included in the guideline text were analyzed.

**Results:**

There is a large discrepancy between the data and the recommendations concerning early antiviral therapy. The studies are either not designed to answer this question or the data is not sufficient to support the arguments. The authors highlight benefits without mention of side effects or other problems. Neither in transmission rates, nor mortality, AIDS events, co-morbidities (hepatitis B and C excluded) or decreasing the risk of malignancy, the data presented support early therapy.

**Conclusions:**

A large discrepancy between the underlining data and the recommendation made by the IAS-USA Panel exists concerning early antiviral therapy.

**Table:** Table 1.

Arguments of committee	Comment	Reference
ART reduces 96% of transmissions	Absolute reduction 0.9 to 0.1 in 100 person years 82% of transmissions in Africa, and 73% of the linked transmissions in o	[1]
Increase of 38% of AIDS events, if started <350 <500 CD4 cells	Absolute increase of AIDS events from 1.78 to 3.63% for a time period of five years, no reduction in mortality	[2]
ART implementation <500 CD4 cell/uL associated with slower disease progression	Absolute decrease of AIDS Events from 1.17 to 0.98/100 person years,	[3]
Higher CD4 cell count associated with decreased risk of AIDS and death in viral suppressed patients up to 500 CD4 cells	Absolute increase of AIDS event or death increase from 0.79 to 1.2 per 100 py	[4]
Lower CD4 nadir leads to poor recovery and increased morbidity and mortality	Only patients with CD4-Nadir <200/uL were included	[5]
41% reduction of serious WHO stage 4 events, pulm. tuberculosis bacterial infection and death treated vs non-treated early	Absolute increase from 2.4 to 3.96/100 py early vs non-early treated	[1]
Patients with CD4 cells >500 has an identical risk of cancer compared to non-HIV	Only comparison 200-499 and >500 CD4 cells, no data on patients 350-499 CD4 cells	[6]
Cross-section studies suggest a benefit of early ART on cardiovascular risk	Only arterial flow mediated dilatation measured. Only comparison between <350 and >350 CD4 cells.	[7,8]
Renal disease increased at lower CD4 counts	Risk of renal disease increases only if CD4 cells fall below 200 cell/uL	[9]
Communities with high ART use have lower rates of new infections	Significant overall, but not if only data from 2004 to 2009 are analyzed, with an increase of ART of 50%. Overall significance	[10]
Decreasing “community viral load” accompanied by decreasing incidence of new HIV infection	No comment	[11]
